# Investigations into SARS-CoV-2 and other coronaviruses on mink farms in France late in the first year of the COVID-19 pandemic

**DOI:** 10.1371/journal.pone.0290444

**Published:** 2023-08-25

**Authors:** Marine Wasniewski, Franck Boué, Céline Richomme, Etienne Simon-Lorière, Sylvie Van der Werf, Flora Donati, Vincent Enouf, Yannick Blanchard, Véronique Beven, Estelle Leperchois, Bryce Leterrier, Sandrine Corbet, Meriadeg Le Gouil, Elodie Monchatre-Leroy, Evelyne Picard-Meyer

**Affiliations:** 1 Lyssavirus Unit, Nancy Laboratory for Rabies and Wildlife, ANSES, Malzéville, France; 2 Wildlife Surveillance and Eco-epidemiology Unit, Nancy Laboratory for Rabies and Wildlife, ANSES, Malzéville, France; 3 G5 Evolutionary Genomics of RNA Viruses, Institut Pasteur, Université Paris Cité, Paris, France; 4 Molecular Genetics of RNA Viruses, CNRS UMR 3569, Institut Pasteur, Université Paris Cité, Paris, France; 5 National Reference Center for Respiratory Viruses, Institut Pasteur, Université Paris Cité, Paris, France; 6 Mutualized Platform of Microbiology, Pasteur International Bioresources Network, Institut Pasteur, Université Paris Cité, Paris, France; 7 Unit of Viral Genetics and Biosafety, Ploufragan-Plouzané-Niort Laboratory, ANSES, Ploufragan, France; 8 DYNAMICURE INSERM U1311 UNIROUEN, Université de Caen, Caen, France; 9 Virology Department, Caen University Hospital, Caen, France; 10 Nancy Laboratory for Rabies and Wildlife, ANSES, Malzéville, France; Federal Medical Centre Abeokuta, NIGERIA

## Abstract

Soon after the beginning of the COVID-19 pandemic in early 2020, the *Betacoronavirus* SARS-CoV-2 infection of several mink farms breeding American minks (*Neovison vison*) for fur was detected in various European countries. The risk of a new reservoir being formed and of a reverse zoonosis from minks quickly became a major concern. The aim of this study was to investigate the four French mink farms to see whether SARS-CoV-2 was circulating there in late 2020. The investigations took place during the slaughtering period, thus facilitating different types of sampling (swabs and blood). On one of the four mink farms, 96.6% of serum samples were positive when tested with a SARS-CoV-2 ELISA coated with purified N protein recombinant antigen, and 54 out of 162 (33%) pharyngo-tracheal swabs were positive by RT-qPCR. The genetic variability among 12 SARS-CoV-2 genomes sequenced from this farm indicated the co-circulation of several lineages at the time of sampling. All the SARS-CoV-2 genomes detected were nested within the 20A clade (Nextclade), together with SARS-CoV-2 genomes from humans sampled during the same period. The percentage of SARS-CoV-2 seropositivity by ELISA varied between 0.3 and 1.1% on the other three farms. Interestingly, among these three farms, 11 pharyngo-tracheal swabs and 3 fecal pools from two farms were positive by end-point RT-PCR for an *Alphacoronavirus* very similar to a mink coronavirus sequence observed on Danish farms in 2015. In addition, a mink *Caliciviridae* was identified on one of the two farms positive for *Alphacoronavirus*. The clinical impact of these inapparent viral infections is not known. The co-infection of SARS-CoV-2 with other viruses on mink farms could help explain the diversity of clinical symptoms noted on different infected farms in Europe. In addition, the co-circulation of an *Alphacoronavirus* and SARS-CoV-2 on a mink farm would potentially increase the risk of viral recombination between alpha and betacoronaviruses as already suggested in wild and domestic animals, as well as in humans.

## Introduction

Soon after the beginning of the COVID-19 pandemic in early 2020, several European mink farms breeding American minks (*Neovison vison*) for fur were found to be infected by SARS-CoV-2. The first infection was detected in April 2020 on a farm in the Netherlands [1] then a second one was identified in Denmark [2]. Infections by SARS-CoV-2 on mink farms were then detected in several other European countries: Italy, Spain, Poland, Greece, Lithuania and Sweden [3]. In Denmark, SARS-CoV-2 spread rapidly on each farm and among mink farms, and was associated with the emergence of a specific variant (called cluster 5) detected in November 2020. At the time, this SARS-CoV-2 variant was believed to have different phenotypic characteristics, including escape from neutralizing antibodies [4, 5]. Moreover, while the primary contamination of the mink farms was due to human infection [6], reverse transmission from minks to humans was detected in both the Netherlands [7] and Poland [8].

In early 2020, four American mink farms of limited size (from less than 1000 to about 15,000 minks according to the French Ministry of Agriculture) were operating in France. These farms were located in different regions. No clinical sign had been observed in minks from these farms since the beginning of the pandemic. The aim of the present study was to investigate the circulation of SARS-CoV-2 and potentially other coronaviruses on the four French mink farms. As mink farming has a seasonal production, with the young adults of the year being slaughtered at the end of the same year, the investigations took place towards the end of 2020 to conduct the most exhaustive survey as possible on the individuals born in 2020. Three different types of samples were collected during the slaughtering period. Blood samples were taken for serologic analyses, swabs were analyzed to detect viral RNA in the upper and lower respiratory tracts, and pooled feces were also analyzed for viral RNA. In addition, Bayesian analysis was used to determine the potential circulation of different lineages on French mink farms.

## Results

### Sample collection

The four American mink farms present in France (named A to D) were investigated. Farms A and D were located in the northwest, in French departments Eure-et-Loir and Orne, respectively. The other two, B and C, were located in the northeast, in Haute-Saône and Meuse, respectively. The farms are separated from each other by at least 130 km and there is no exchange of animal or equipment between them.

A total of 1912 minks born in 2020 were sampled for blood, with a minimum of 60 animals per building, and 1643 pharyngo-tracheal swabs were collected. The characteristics of each farm and of the sampling are presented in Table 1.

**Table 1:** Identification of mink farms, number of blood samples by sampling route, swab samples and fecal samples collected in November 2020 in France from adult minks of the year. **A “**scanstar” is an outdoor animal facility hosting minks.

Farm	Minks present on the farm	Nb. of scanstars	Nb. of scanstars sampled from housings for minks slaughtered for fur	Blood samples		Pharyngo-tracheal swabs	Nb. of scanstars sampled from housings for live minks kept for breeding	Fecal samples (nb. of pools collected in each scanstar)
				Intra-cardiac puncture	Retro-orbital			
**A**	3800	3	3	179		162	0	0
**B**	950	2	2	120		120	0	0
**C**	7900	15	9		403	395	6	56^a^
**D**	10,850	26	20	1210		1230	6	34^b^
**Total**	23,500	47	34	1912		1643	13	90

^a^ number of fecal samples collected / number of minks sampled: 1–10 / 2–5 animals

^b^ number of fecal samples collected / number of minks sampled: 4–7 / 10 animals

### Detection of SARS-CoV-2 antibodies (ELISA and seroneutralization test)

All mink blood samples were tested by a SARS-CoV-2 ELISA coated with a purified N protein recombinant antigen. All samples with a doubtful or positive result were systematically tested again using a seroneutralization assay to confirm or overturn the ELISA results.

For farm A, where evidence of an ongoing infection was noted (SARS-CoV-2 RNA was found in pharyngo-tracheal swabs), we randomly selected 16 samples among the positive sera to be tested by a seroneutralization assay. For farms B, C and D, samples that were negative by ELISA were also tested by a seroneutralization assay as negative controls.

The results of the ELISA are shown in Table 2. The percentage of seropositivity varied between 0.5 and 1.2% in non-infected farms and reached 96.6% in the infected farm (A). For farm A, the S/P% values of positive samples ranged from 62.3 to 492.6 with a mean value equal to 307.9 (N = 173).

**Table 2:** Serologic results (positive/doubtful/negative) obtained by an ELISA on mink serum samples collected in November 2020 on the four mink farms in France.

Farm	Samples	Positive	Doubtful	Negative	Percentage of positives
**A**	179	173	1	5	96.6
**B**	120	1	0	119	0.8
**C**	403	1	1	401	0.3
**D**	1210	13	1	1196	1.1

The samples found positive or doubtful from farms B, C and D, as well as a few randomly selected positive samples from farm A, were tested by a seroneutralization assay (Table 3).

**Table 3:** Serologic results obtained using the seroneutralization assay on ELISA-positive/-doubtful mink serum samples collected in November 2020 on the four mink farms in France.

Farm	Samples	Positive	Negative	Percentage of positives
**A**	16	16	0	100
**B**	1	0	1	0
**C**	2	0	2	0
**D**	14	0	14	0

For the three non-infected farms, no SARS-CoV-2 neutralizing antibody was detected by a seroneutralization assay in the positive and doubtful sera obtained by an ELISA (Table 3). However, for farm A, SARS-CoV-2 neutralizing antibodies were detected in the 16 ELISA-positive samples. Neutralizing titers ranged from 81 to above 2239, confirming that the virus was circulating on this farm.

### Detection of SARS-CoV-2 viral RNA in pharyngo-tracheal swabs

The TaqMan RT-qPCR analysis of pharyngo-tracheal swabs revealed that farm A was experiencing an ongoing SARS-CoV-2 outbreak: of the 162 minks tested from farm A, SARS-CoV-2 RNA was detected in 54 swabs at the date of sampling (Table 4). Of the 54 positive samples, 33 of them showed low levels of viral RNA (Ct value > 32, which corresponds to approximately 0-5copies/μL of RNA). The 21 positive minks for SARS-CoV-2 RNA (Ct values ranging from 18.8 to 31) were found positive with RNA titers ranging from 10 to 3.79E+04 copies/μL of RNA.

**Table 4:** SARS-CoV-2 RNA detection in pharyngo-tracheal swabs from farm A.

Building number	Detected^a^	Not detected	NI	Total
**1**	22	31	2	55
**2**	17	32	3	52
**3**	15	37	3	55
**Total**	54	100	8	162

^a^ Detection of SARS-CoV-2 RNA with Ct values < 36 and two replicates positive / 2

**NI**: not interpretable (detection in one replicate out of two replicates tested with C_T_ values varying between 30 and 36)

**Negative**: C_t_ values >39

No SARS-CoV-2 RNA was detected in samples from the other three farms (n = 1481 samples tested).

### SARS-CoV-2 genomes

**Fig 1:**
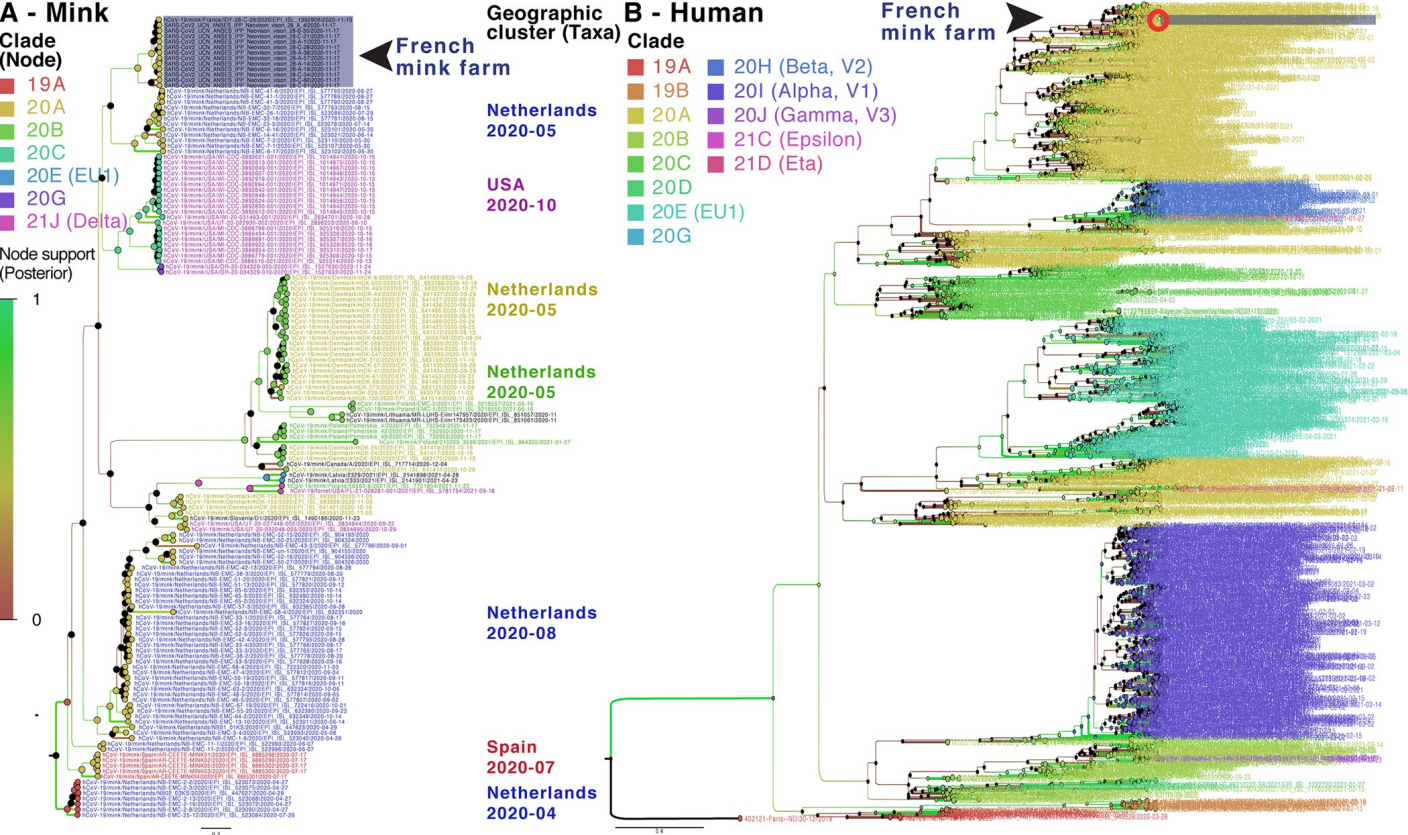
Bayesian phylogenies on a mix of representative sets of SARS-CoV-2 sequences available in GISAID. SARS-CoV-2 sequences detected on the mink farm in France are highlighted in purple. **A. Bayesian phylogeny of all SARS-CoV-2 genomes from minks over the study period.** Node and taxa labels are colored according to nextstrain (https://nextstrain.org) clade classification and main geographic clusters, respectively. **B. Bayesian phylogeny of human-derived SARS-CoV-2 genomes collected in France and mink-derived SARS-CoV-2 genomes described in this study.** A diversity-optimized dataset was obtained by collecting and filtering complete, high-quality genomes of SARS-CoV-2 detected in France (of human and mink origin). Taxa are colored according to the nextstrain clads classification.

SARS-CoV-2 RNA samples with Ct values less than 31 (12.5–3.1E+02 copies/μL of RNA) were subjected to full genome analysis. Nearly complete viral genome sequences (coverage > 99.4%, average depth > 15 000, maximum depth > 100 000) were obtained from each of the 12 nucleic acid extracts that were positive for SARS-CoV-2 by an RT-qPCR (Ct values of 26.2–30). An additional extract, weakly positive by an RT-qPCR, yielded partial sequences corresponding to different regions of the genome. All twelve genomes (GISAID under numbers EPI_ISL_1392906 & EPI_ISL_10036487–97) were classified as 20A based on a panel of mutations compared with reference sequence NC_045512 and a Nextclade analysis (S1 Fig, S1 Table). A Bayesian phylogenetic analysis using all high-quality mink-derived SARS-CoV-2 genomes available in the GISAID database clustered SARS-CoV-2 from the mink farm in France together in a monophyletic clade supported by a significant node value (posterior probability) and a relatively long branch (Fig 1). This clear clustering does not link the SARS-CoV-2 from the mink farm in France to any another mink farm.

**Fig 2:**
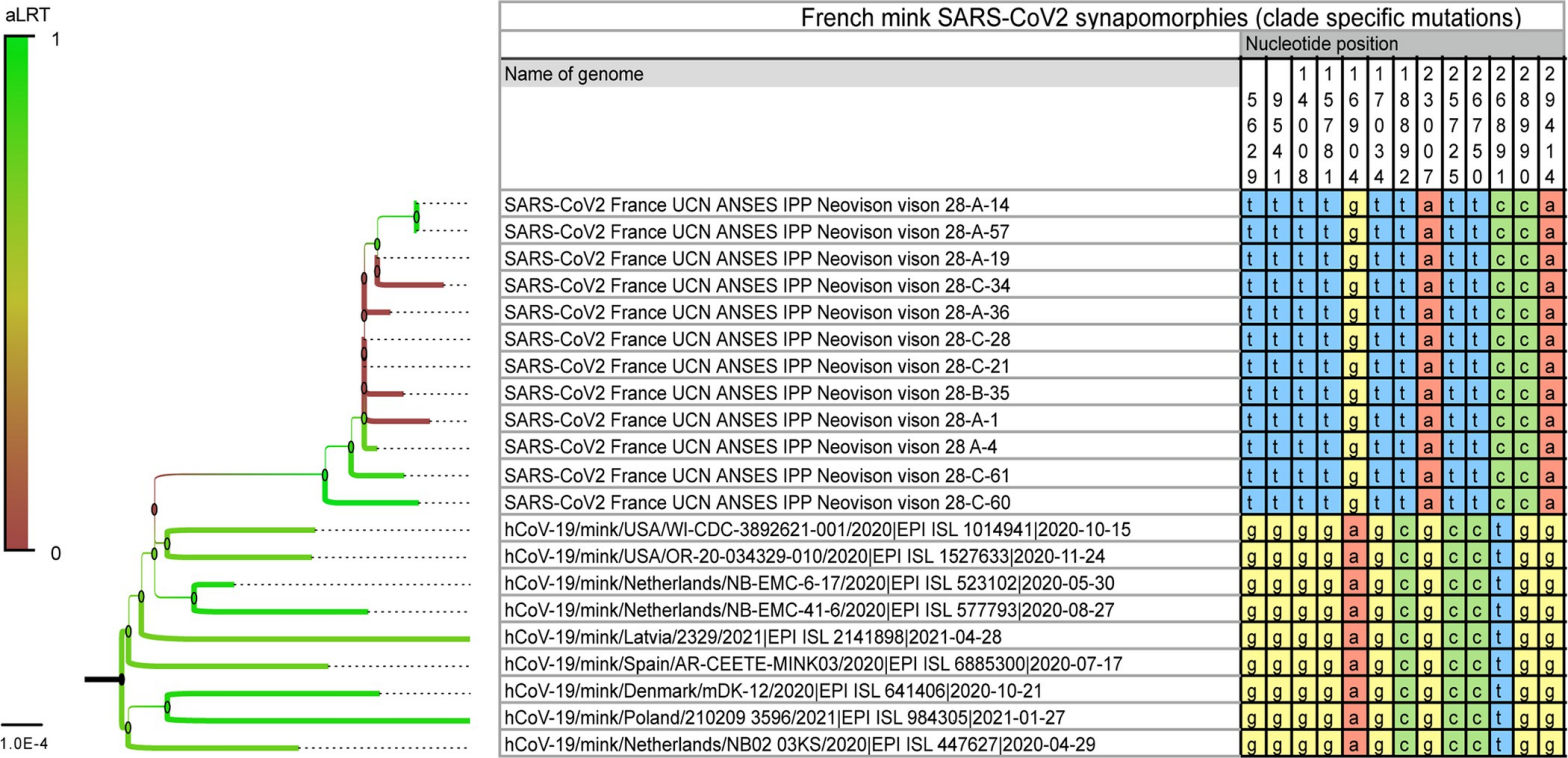
SARS-CoV-2 synapomorphies in American minks from a mink farm in France with the 13 SNPs specific to the clade formed by the mink SARS-CoV-2 in France compared with other SARS-CoV-2 from mink located elsewhere. SNPs are represented with the nucleotide positions on the SARS-CoV-2 genome, and in yellow for G, red for A, green for C and blue for T. Branches are colored according to the bootstrap values.

Moreover, 13 single nucleotide polymorphisms (SNPs) were specific to this clade, unlike other SARS-CoV-2 clades detected in mink (*Neovison vison*) worldwide at the time of data collection and phylogenetic analyses based on 821 mink SARS-CoV-2 genomes retrieved on January 5, 2022 (Fig 2; S1 Table). Among the SNPs that differentiate mink SARS-CoV-2 sequences from France from other available mink SARS-CoV-2 sequences, four induce an amino-acid change (V676L, K1141R, E1184D in the Orf1b and S477N in the spike). Within this monophyletic clade, genetic variability was observed among SARS-CoV-2 genomes from the French farm (including non-silent mutations), indicating the co-circulation of several variants in this setting at the time of sampling (S1 and S2 Tables). A Bayesian phylogenetic analysis using the GISAID data for 2020 (437 human SARS-CoV-2 genomes in a diversity-optimized matrix based on 99.875% pairwise nucleotide identity cutoff from 42,700 genomes) showed that the SARS-CoV-2 genomes detected on the mink farm in France nested within the 20A clade, together with SARS-CoV-2 genomes from humans sampled at the same period (S2 Fig). In addition, the non-silent mutation in S (S477N) that differentiates these mink SARS-CoV-2 genomes from those of SARS-CoV-2 from mink elsewhere, was also observed in SARS-CoV-2 in samples from humans in France at the same period (S3 Table and S2 Fig).

### Detection of an *Alphacoronavirus* in pharyngo-tracheal swabs and feces

We analyzed the pharyngo-tracheal swabs and feces for the presence of RNA coronaviruses by end-point RT-PCR targeting the pol gene. The end-point RT-PCR analysis of pharyngo-tracheal swabs revealed that two out of three farms were positive for RNA coronaviruses distinct from SARS-CoV-2. Of 236 swab samples tested, 11 minks were positive for *Alphacoronavirus* RNA: Two samples were from farm C and nine from farm D (Table 5). No coronavirus was detected in swabs from farm B. Farm A samples were not analyzed for the presence of the A*lphacoronavirus* genome. A total of 90 pools of feces (56 from farm C and 34 from farm D) were simultaneously tested by an RT-PCR targeting the pol gene and a beta-actin RT-PCR for investigating the presence of RNA inhibitors. The beta-actin housekeeping gene RNA was not detected in 46.6% of the fecal samples tested (n = 42/90*100). Farm C had a higher proportion of unexploitable samples (55.4% = 31/56*100) than farm D (32% = 11/34*100). Of 48 exploitable fecal pools, three were positive for *Alphacoronavirus* RNA: one from farm C and two from farm D.

**Table 5:** Detection of α-CoV in pharyngo-tracheal swabs and feces in SARS-CoV-2-negative mink farms in France.

		**SARS-CoV-2 RNA RT-qPCR on**	**CoV RT-PCR of pol gene on**	
		**Pharyngo-tracheal swabs**	**Pharyngo-tracheal swabs** ^**a**^	**Pools of feces** ^**a**^
**Farm**	**Nb of buildings**	**Positive/tested (%)**	**Positive/tested (%)**	**Positive/”exploitable” (%)**
**B**	2	0/120 (0)	0/28^a^ (0)	Not tested
**C**	9	0/395 (0)	2/65^a^ (3.1)	1/25 ^a^^,^^b^ (4)
**D**	20	0/966 (0)	9/143^a^ (6.3)	2/23 ^a^^,^^c^ (8.7)

^a^ Randomized collection from different farm buildings

^b^ For farm B, of 56 pools tested, 31 samples tested negative for the presence of the beta-actin gene and were then considered as “unexploitable”. The prevalence was calculated as follows: samples that had tested positive for the presence of coronavirus RNA / total of “exploitable” samples *100.

^c^ For farm C, of 34 pools tested, 23 samples tested positive for the presence of the beta-actin gene and 11 tested negative (these 11 were then considered “unexploitable” for calculating the prevalence).

**Fig 3:**
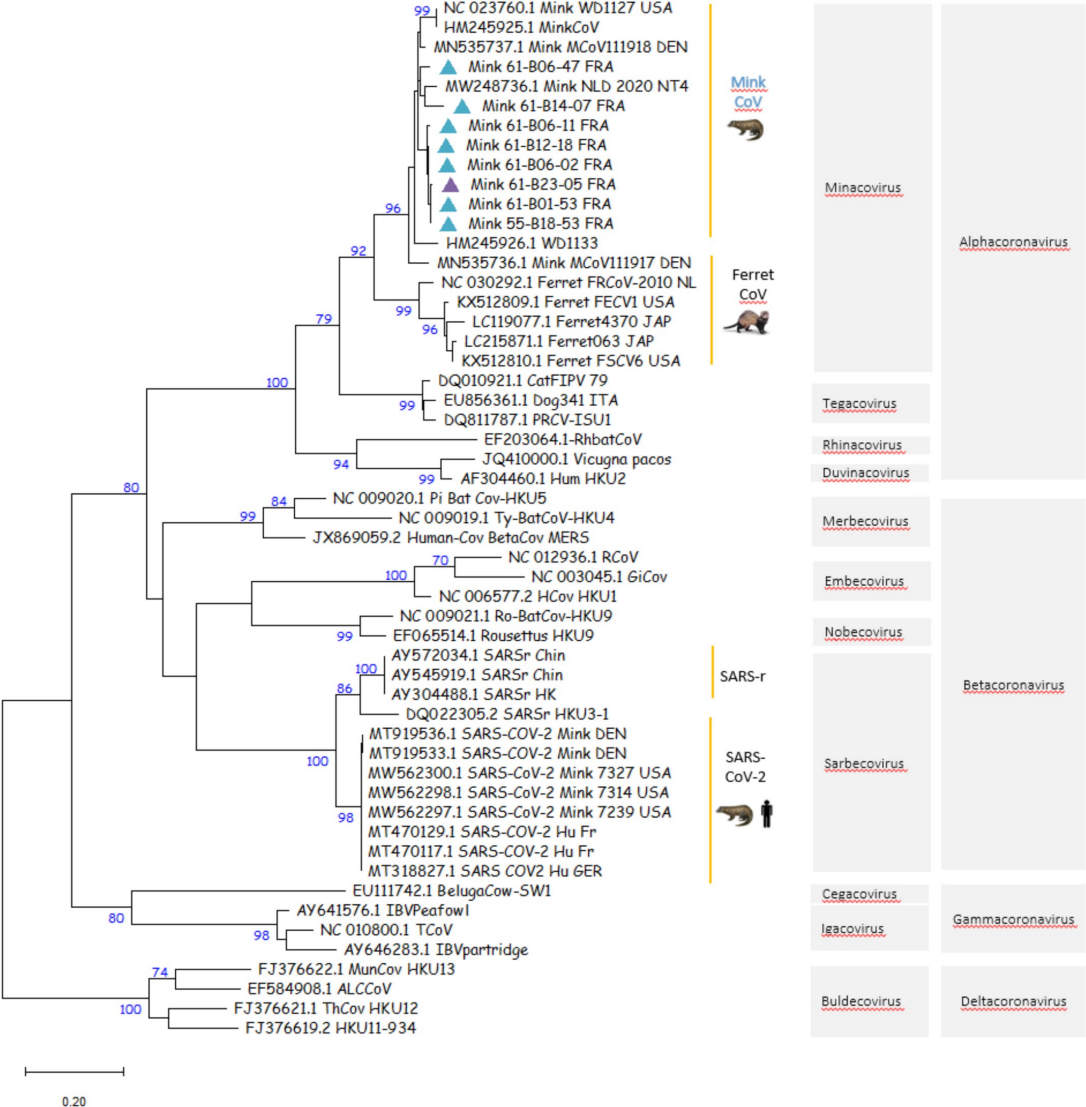
Maximum likelihood (ML) phylogeny inferred with eight mink coronavirus consensus sequences from France and 45 representative GenBank sequences including alphacoronaviruses (n = 25), betacoronaviruses (n = 20) with mink and human SARS-CoV-2 sequences, gammacoronaviruses (n = 4) and deltacoronaviruses (n = 4). Bootstrap values above 70% were considered as statistically significant. Pharyngo-tracheal swabs and feces infected by the mink coronavirus are represented on the tree by a turquoise or purple triangle, respectively. The eight partial pol gene consensus sequences included in the ML tree are accessible in GenBank under accession numbers: Mink_61-B14-07_FRA: ON985270; Mink_61-B12-18_FRA: ON985271; Mink_61-B06-47_FRA: ON985272; Mink_61-B06-11_FRA: ON985273; Mink_61-B06-02_FRA: ON985274; Mink_61-B01-53_FRA: ON985275; Mink_55-B18-53_FRA: ON985276; Mink_61-B23-05_FRA: ON985277.

Of the 14 samples that tested positive by an RT-PCR for the pol gene, five were weakly positive and then were either not subjected to or failed the SANGER sequencing. Sequence analyses of PCR products (n = 9) showed that all the SANGER-sequenced samples (from seven pharyngo-tracheal swabs and two fecal pools) contained alphacoronaviruses. Of the nine nucleotide consensus sequences, eight were included in the phylogenetic analysis, all of them grouped within *Alphacoronavirus*, *Minacovirus* subgenus (bootstrap = 95), close to the mink coronavirus (previously named ferret coronavirus–Fig 3). The ninth sample, 61-B23-2, was not included in the phylogeny due to the poor quality of the nucleotide sequence obtained by SANGER sequencing and the fact that we were unable to obtain the consensus sequence for this sample. BLAST analysis of the forward sequence nonetheless showed that sample 61-B23-2 was close to both mink coronavirus 1 MN535737 isolated in Denmark in 2015 and an *Alphacoronavirus* isolated from a mink in China in 2016 (MF113046). Close nucleotide similarities were observed for the Pol gene sequences from farm C (n = 1) and farm D (n = 8). A BLAST search showed that the nine strains shared between 94.6% and 96.3% identity at the nucleotide level with the mink coronavirus 1 (MN535737) strain isolated in Denmark in 2015.

Of the three fecal samples positive for the coronavirus pol gene, the most positive sample (61-B23-05) was subjected to high-throughput sequencing (HTS). The 61-B23-05 extract yielded many gaps throughout the genome, with partial sequences (i.e. 706, 639, 597, 445, and 206 nucleotides) corresponding to five different regions of a mink strain coronavirus 1 MCoV1/11918-1/DK/2015 (MN535737.1). A BLAST analysis of the five partial sequences showed 89–95% identity with MN535737.1.

### Detection of the mink *Caliciviridae* RNA genome in pharyngeo-tracheal samples

Deep sequencing of viral RNA extracted from pharyngeo-tracheal swab samples 61-B06-11 and 61-B12-04 mainly generated bacteria or Mustelidae host-originating reads; only 0.06% to 0.7% of the reads were identified as viral sequences with, for sample 61-B12-04, 75% of the reads identified as mink *Caliciviridae*. None of the reads obtained for these two samples corresponded to *Alphacoronavirus* sequences. After the Spades assembly of the 61-B12-04 reads, one contig (4320 nucleotides long) was identified as a *Caliciviridae* sequence with 95% identity with the *Caliciviridae* strain Mink/China/2/2016 (MF67785). Several reads were identified as mink *Caliciviridae* in the two samples. The whole genome size for the two samples was of 8427 nt in length and consisted of three open reading frames and two untranslated regions (5’ and 3’) 13 and 103 nt long, respectively. ORF1 ranged from nt 14 to nt 5851 (encoding a polyprotein of 1946 aa), ORF2 from nt 5857 to nt 7899 (681 aa), and ORF3 from nt 8130 to nt 8306 (59 aa). The alignment of truncated sequence 61-B12-04 (GenBank Number OP485683) with the MF677852 mink *Caliciviridae* strain from China showed an amino-acid identity of 99.1% across the whole genome), with 99.4% for ORF1, followed by 98.4% and 98.3% for ORF2 and ORF3, respectively. Finally, BLAST analysis of the 61-B06-11 and 61-B12-04 sequences showed > 94.4% (E value = 0.0; Score = 8167; number of hits = 5; 4982/5279*100) and 96% (E value = 4e-103; Score = 392; number of hits = 5; 230/239*100) of nucleotide identity with the mink *Caliciviridae* strain MF677852 from China, respectively.

## Discussion

This study describes the investigations into SARS-CoV-2 on the four mink farms operating in France late in the first year of the COVID-19 pandemic. The small number of farms and animals per farm made it possible to implement a robust transversal study that could detect a minimum prevalence of 5%. On one farm, minks were found to be infected with SARS-CoV-2, with a high serologic prevalence (above 96%) and SARS-CoV-2 RNA was detected in 30% of the sampled minks. The infection had spread throughout the three farm buildings. No clinical sign nor suspicious mortality was observed by the breeder, suggesting a mild outbreak that could easily have been missed in the absence of investigations [2, 9, 10].

Despite some variability, all 12 SARS-CoV-2 genome sequences obtained belonged to the 20A clade, according to Nextclade and two Bayesian phylogenetic reconstructions. Given the genetic relationship between the SARS-CoV-2 sequenced from the mink farm in France and the SARS-CoV-2 responsible for the concomitant epidemic wave of 20A viruses in humans, as well as the previous description of the zoonotic circulation of SARS-CoV-2 between humans and minks in the Netherlands, reverse zoonosis appears to be the main hypothesis to explain the SARS-CoV-2 circulation detected in the minks on this farm. In addition, comparison with other SARS-CoV-2 sequences from minks worldwide clearly shows the monophyly of this mink clade found in France. The significant genetic distance to viruses found in other countries is supported by 13 mutations specific to this clade. These analyses reinforce the hypothesis of a local transmission from humans to explain the origin of the circulation in minks in the present study. Among these mutations specific to this mink clade, four were non-silent and one, resulting in the S477N change in the spike, was also present in several genomes sequenced from humans during the same period in France. Despite the substantial genetic variability observed here in mink SARS-CoV-2, data were not discriminant enough to test the monophyly of this mink clade with sufficient confidence when analyzed with all the human SARS-CoV-2 samples available from the region and collected during the same period. Bayesian reconstruction shows apparent paraphyly of the mink clade found in France, with some internal human SARS-CoV-2 sub-clustering, but these nodes are not significant (low posterior probabilities). The dataset does not clearly reveal whether one or more reverse-zoonotic events occurred. However, the relative genetic identity of genomes detected on the farm supports the hypothesis of contamination relatively shortly before the sampling, with viral circulation probably within the previous 3 months. Indeed, this short a period is insufficient to allow diversification into several clearly distinct lineages as the accumulation is around two mutations a month on average [11]. Many minks can be infected within a short time: Hammer et al. [9] described an increase in prevalence from 4% to 97% in eight days among minks on a farm in Denmark. The detection of SARS-CoV-2 RNA in pharyngo-tracheal swabs with Ct values varying between 18.8 and 38.4 was the sign that infection was recent in a few minks, especially for those with low Ct values. Indeed, after experimental infection, viral RNA can be detected in the upper respiratory tract of exposed minks from 2 dpi to 17 dpi [12]. As the detection of SARS-CoV-2 on farm A induced immediate control measures (culling of all minks), it was not possible to monitor the genomic evolution of the virus in minks over time or to test any potential spill-back to humans.

No SARS-CoV-2 contamination was detected on the other three farms, but less than 1% of the samples on farms B and C, and 1% on farm D were positive by an ELISA, despite a negative seroneutralization test. The specificity of the ELISA test in minks is very high (close to 99%), which is better than in humans (where the specificity is evaluated between 92.5 and 98.8%) [13, 14]. On farms C and D, in northeast and northwest France, respectively, we detected an *Alphacoronavirus* which corresponded to a mink coronavirus sequence, found in both pharyngo-tracheal swabs and feces. Mink coronavirus infection is associated with epizootic gastroenteritis (ECG) in minks when in combination with several enteric viruses, but it can also be asymptomatic [15]. As ECG is of economic concern, several authors in different countries have studied the disease’s characteristics (distribution, lesions, etc.) [15, 16]. Considering the ECG frequency found in these studies, mink coronavirus infection in fur minks does not appear to be rare, but to our knowledge, few data exist on prevalence. However, the potential circulation of an *Alphacoronavirus* at the same time as a very active SARS-CoV-2 outbreak on a farm would considerably increase the risk of viral recombination. The recombination of alpha- and betacoronaviruses has been already described in wild and domestic animals as well as in humans [17–19]. Such an event could result in an evolutionary jump and may generate a recombinant with an unpredictable phenotype and fitness that may foster the emergence of a novel coronavirus. This potential issue should be seriously considered in countries where mink farms (or farm breeding of other small carnivores) are insufficiently monitored and where there is no preventive culling of animals on farms positive for SARS-CoV-2.

Finally, when attempting to obtain the whole genome sequence of mink coronavirus from swab samples that were positive for *Alphacoronavirus* by an end-point RT-PCR, a nearly complete genome of a mink *Caliciviridae* was obtained by HTS. The *Caliciviridae* sequence was predominant in our two samples over that of the mink coronavirus for which no sequence was obtained. The failure by HTS to detect mink coronavirus was disappointing but can be explained by the very low load of coronavirus RNA, which was only detected after a nested PCR. As a result, coronavirus reads were hidden by the high proportion of bacteria and host (mustelidae) reads accounting for ~99% of the reads in this sample. HTS sensitivity is known to be much lower than that of PCR, and especially nested PCR. Similar *Caliciviridae* have already been sequenced in minks in the USA [20] and China [21]. The associated clinical signs are not yet fully identified; some authors described no clinical signs [22] or diarrhea [22] or hemorrhagic pneumonia [20]. Beside the results obtained on coronaviruses, our study on the French mink farms also shows that a number of potentially pathogenic agents were silently circulating. This also seems to be the case on other farms [22]. These infections could modify the symptomatology of SARS-CoV-2 infection in minks, which has been reflected in more or less acute clinical signs depending on the farms in the different countries affected.

## Materials and methods

The four American mink farms in France (named A to D) were all in rural areas isolated from human habitations. They were small family farms managed by one person, except farm D where external workers were regularly employed. On each farm, minks were housed in wire mesh cages placed in scanstars, each of these unwalled housekeeping buildings consisting of two rows of several dozen cages. A surrounding fence assured protection from intrusion into the farm premises. During the investigations in November 2020, each cage housed two adult minks. Each cage was equipped with automatic water distribution, and food was distributed every two days. According to the farmers’ declaration, no material was shared between farms and no animals were exchanged.

Before slaughtering, the four mink populations consisted of 3800 minks on farm A, 950 on farm B, 7900 on farm C and 10,850 on farm D, spread over 3, 2, 15 and 26 housekeeping buildings, respectively.

### Mink sampling and sample collection

Each scanstar was considered an independent epidemiological unit. Sixty animals were sampled in each to enable sensitive detection, assuming a minimum apparent prevalence of 5%, with a 95% confidence interval.

Minks were sampled during the slaughtering period (10 to 26 November 2020). Blood was sampled from freshly euthanized mink carcasses by intra-cardiac puncture on farms where the animals were pelted on-farm (farms A, B, and D) or by retro-orbital sampling on farm C where animals were stored intact before being sent elsewhere for pelt processing. Blood samples were stored at 4°C before being transferred to the laboratory. Samples were allowed to clot and were then centrifuged (1000g, 15 min) to obtain serum. Sera were stored at -20°C until serologic testing. Sera were heat-inactivated at 56°C for 30 min before the seroneutralization assay.

Pharyngo-tracheal swabs were collected from mink carcasses by the retrotracheal route on farms A, B, and D, and by the oropharyngeal route on farm C. The swabs were immediately kept in a volume of 500 μL of cell culture medium (DMEM) supplemented with 1% of antibiotics (a mix of penicillin, streptomycin and amphotericin B) before being frozen in liquid nitrogen to ensure viral integrity. The samples were transferred to the laboratory and stored at < -70°C until RNA extraction.

The blood sample collection was complemented with fecal samples from breeding animals on farms C and D. Indeed, on these two farms, some scanstars housed the animals that would serve as breeding stock and had not therefore been slaughtered. It was not possible to take blood samples or tracheal swabs from these minks. Pools of feces were collected from six individual scanstars on farms C and D, leading to a total of 56 and 34 pooled samples for the two farms, respectively. Samples were stored at < -70°C until RNA extraction.

### SARS-CoV-2 isolation

SARS-CoV-2 strain UCN19 was amplified on cells as described previously [23] and used on passage 2 for the seroneutralization assays.

### Enzyme -linked immunosorbent assay (ELISA)

The enzyme-linked immunosorbent assay provided by IDVet (ID Screen®ELISA, SARS-CoV-2 Double Antigen Multi-species) was used in this study as a screening tool to detect the presence of antibodies in mink sera. This ELISA is a double antigen ELISA for the detection of antibodies directed against the nucleocapsid of SARS-CoV-2 in animal serum, plasma or whole blood. The samples were tested according to the manufacturer’s recommendations and as previously described [24, 25]. Briefly, 25 μL of each serum sample was placed in the microplate and diluted 1:2 in sample diluent. Microplates were incubated for 45 minutes at 37°C+/-2°C. Five washings were performed after incubation, then 100 μL of the conjugate (a purified recombinant N protein antigen labeled with horseradish peroxidase) was added to each well. The microplates were incubated for 30 min at 21°C +/-5°C. Five washings were performed to remove the unbound conjugate. The presence of the complex antibodies/conjugate was revealed by adding 100 μL of TMB (Tetramethylbenzidine) chromogen solution to each well. The microplates were incubated in the dark for 20 min at 21°C +/-5°C. The enzymatic reaction was stopped by adding 100 μl of a stop solution. The microplates were read at 450 nm. Positive and negative controls provided by the manufacturer were used to validate each test plate.

The conditions of validation described by the manufacturer were implemented to validate the tests and to interpret the results obtained for the different samples. The “Sample/Positive” ratio was calculated as follows and expressed as a percentage (S/P%):

$$S/P\%=\frac{\mathrm{ODSample}‐\mathrm{ODNC}}{\mathrm{ODPC}‐\mathrm{ODNC}}\times 100$$

where ODNC is the optical density of the negative control and ODPC is the optical density of the positive control and ODSample is the optical density of the sample. According to the cut-off determined by the manufacturer, three kinds of results could be obtained:

- If S/P% is below or equal to 50%, the sample is considered negative
- If S/P% is between 50% and 60%, the sample is considered doubtful
- If S/P% is above or equal to 60%, the sample is considered positive

### Seroneutralization assay

Briefly, 24 hours before starting the seroneutralization assay, 200 μL of VERO E6 cell suspensions in Dulbecco’s Modified Eagle Medium (DMEM) containing 10% FCS (fetal calf serum) and 1% antibiotics (penicillin/streptomycin) were added to each well of a 96-well microplate, representing 20,000 cells per well.

Each serum sample as well as positive and negative internal controls were distributed in two consecutive wells of 96-well microplates, and then serially diluted with a dilution step of 1 to 3 in DMEM containing 10% FCS and 1% antibiotics (penicillin/streptomycin). Then 50 μL of SARS-CoV-2 virus diluted in medium containing around a 50% tissue culture infective dose (TCID50) of 100 per 50 μL (checked by back-titration during the seroneutralization assays) were added to each well containing samples and internal controls. The plates were incubated at 37°C/5% CO_2_ for 1 h to allow neutralization complexes to be formed between the neutralizing antibodies and the virus. At the end of the incubation, the supernatant fluid was removed from each well of the plates containing VERO E6 cell suspensions and immediately 100 μL of the mix containing either the virus and serially diluted samples or the virus and controls were transferred to individual wells on the cell layer. The microplates were incubated at 37°C in a humid chamber containing 5% CO_2_, at least 3 days post-infection. The plates were then qualitatively read according to an “all or nothing” scoring method for the presence of viral cytopathic effect (CPE). The neutralization titers were assigned to each serum based on the highest dilution that prevented a discernible cytopathic effect.

### RNA extraction

#### Pharyngo-tracheal swabs

Viral RNA was extracted from 140 μL of pharyngo-tracheal swabs medium. Viral RNA extraction was performed using the Qiagen Viral RNA mini kit according to the manufacturer’s instructions (Qiagen, Les Ulis, France), with minor modifications. To inactivate samples that were potentially infectious due to SARS-CoV-2, a volume of 15 μL of Triton X-100 (MP Biomedicals, Illkirch, France) was added to 560 μL of AVL viral lysis buffer (Qiagen, Courtaboeuf, France) for each sample tested. RNA was eluted in a final volume of 60 μL and stored at < -70°C. A negative RNA extraction control was performed for each set of 24 samples tested.

#### Fecal samples

100 mg of fecal sample was placed in a lysing matrix E tube containing beads (MP Biomedicals Germany Gmbh, Eschwege, France) and filled with 1 ml of CTAB buffer (Promega France, Charbonnières les Bains, France). Tubes were placed in a shaking heat block at 65°C, vigorously vortexed for 1 min and mixed with 40 μL of Proteinase K Solution (Promega France, Charbonnières les Bains, France). After incubation at 70°C for 10 min, the lysates were ground for 30 sec at 7.0 m/s six times in a Fast Prep-24 ^TM^ 5G bead beater (MP Biomedicals Germany Gmbh, Eschwege, France) then centrifuged at 10,000 x g for 5 min. A volume of 300 μL of clear lysate was transferred to a tube containing 300 μL of lysis buffer. RNA was extracted with the Maxwell RSC PureFood GMO and Authentication kit using a Maxwell RSC instrument (Promega France, Charbonnières les Bains, France), according to the manufacturer’s instructions. RNA was eluted in a final volume of 100 μL and stored at < -70°C.

Negative (non-template control) and positive (hedgehog betacoronavirus) controls (RNA extraction control) were performed for each set of 16 samples tested.

### TaqMan RT-qPCR of the E gene using specific SARS-CoV-2 primers

A TaqMan RT-qPCR was performed as previously described [23]. Coronavirus primers (E_Sarbeco_F (forward): 5’-ACAGGTACGTTAATAGTTAATAGCGT and E_Sarbeco_R (reverse): 5’-ATATTGCAGCAGTACGCACACA) and probe (E_Sarbeco_P1: 5’-FAM-ACACTAGCCATCCTTA CTGCGCTTCG-BHQ-1) targeting the envelope protein gene (E gene) were used for the study [26]. Primers and probe were provided by Eurogentec (Angers, France). TaqMan RT-qPCR assays were performed in a total volume of 25 μL containing 2.5 μL of RNA sample, 12.5 μL of 2x QuantiTect Probe RT-PCR Master Mix, 1 μL of 25 mM MgCl2 (Invitrogen), 4.5 μL of RNase-free water, 2 μL each of forward and reverse primer (10 μM), 0.25 μL of probe (10 μM), and 1 μL of QuantiTect RT Mix. All TaqMan RT-qPCR assays were performed on the thermocycler Rotor Gene Q MDx (Qiagen, Courtaboeuf, France). Amplification was carried out according to the following thermocycling conditions: 50°C for 30 min for reverse transcription, followed by 95°C for 15 min and then 45 cycles of 94°C for 30 s, 55°C for 30 s and 72°C for 30 s. Negative and positive controls were included in each RT-qPCR assay.

The titer of SARS-CoV-2 RNA as number of copies/μL was determined by testing six 10-fold dilutions (i.e., 1.05.10^8^ to 1.05.10^3^ genome copies/mL of a quantitative synthetic RNA from SARS-CoV-2 (BEI Resources). A threshold setting (Ct) of 0.03 was used as the reference threshold for each RT-qPCR assay. The efficiency, slope and correlation coefficient (R^2^) were calculated by the Rotor Gene software. All reactions were carried out as technical duplicates. A cut-off > 36 was defined for negative results and between 32 and 36 for weak positive results (samples with late C_T_ values).

### End-point RT-PCR of the pol gene in coronaviruses using conserved primers

Coronavirus RNA was detected by amplifying a 438-bp fragment of the RNA-dependent RNA polymerase (pol) gene of coronaviruses using the following degenerated primers: PanCoV pol 15197 (forward): 5’-GGTTGGGAYTAYCCWAARTGTGA, PanCoV pol 15635 (reverse): CCATCRTCMGAHARAATCATCATA designed by [27]. The end-point RT-PCR was performed in a two-step RT-PCR with synthesis of cDNA from the extracted total RNA followed by a touchdown PCR.

The cDNA was synthesized by reverse transcription of 5 μL of RNA extracted from tracheal swabs and fecal samples using 0.25 μL of hexanucleotide primers (0.2 μG/μL) (Thermo Fisher Scientific, Dardilly, France) and an RT Maxima H Minus cDNA synthesis kit (Invitrogen, Thermo Fisher Scientific, Dardilly, France) according to the manufacturer’s instructions. cDNA synthesis was performed for 10 min at 25°C, 30 min at 50°C followed by a final step of 5 min at 85°C. cDNA was stored at < -70°C.

A PCR was performed in a final volume of 25 μL containing 3 μL of cDNA, 2.5 μL of 10X PCR Buffer (Invitrogen, Marseille, France), 0.75 μL of MgCl_2_ (50 mM), 1 μL of dNTPs (10 mM) and 0.5 μL of Platinum Taq DNA polymerase (5 U/ml) (Invitrogen, Marseille, France) and 1 μL of forward and reverse primer (20 μM). The PCR was amplified for 2 min at 94°C, with 11 cycles of 30 s at 94°C, a 1° touchdown decrease of the annealing temperature from 60° to 50°C, 90 s at 72°C, then 40 cycles of 30 s at 94°C, 45 s at 50°C and 90 s at 72°C followed by a final extension step of 10 min at 72°. Negative and positive controls were included in each RT and PCR assay.

The beta-actin gene was amplified for each fecal sample with the forward (5’-CGATGAAGATCAAG/ATCATTGC-3’) and reverse (5’-AAGCATTTGCGGTGGAC-3’) primers, using the same methodology to confirm the absence of PCR inhibitors in samples. The amplified products were analyzed by electrophoresis on a 2% agarose gel stained with a SYBR safe solution at a final concentration of 1/10,000 then photographed.

### Sequencing of PanCoV amplicons, alignment of sequences and phylogeny

The positive PCR products were sequenced in both directions by Eurofins Genomics (Germany) with the same specific primers used in the PCR.

A dataset of sequences was constituted with eight sequences from this study (seven pharyngo-tracheal swabs and one fecal sample) and 41 referenced sequences including representative sequences of the genus *Alphacoronavirus (n = 25)*, *Betacoronavirus (n = 20)*, *Deltacoronavirus* (n = 4) and *Gammacoronavirus (n = 4)*. A multiple alignment of partial pol sequences (positions 14113 to 14536 compared with the reference genome of a *Minacovirus* Strain MW248736) was carried out with Mega v10.1.8 [28]. A phylogenetic tree was constructed using the Ml method (GTR model). Node robustness was estimated using the bootstrap method with 1000 iterations. The consensus sequences of the partial genome of the pol gene are accessible in GenBank under accession numbers ON985270 to ON985277.

### Next-generation sequencing from saliva swabs

#### Non-specific nanopore sequencing

Nanopore non-specific sequencing and bioinformatic analyses were performed in the virology department / university of Caen and data were analyzed using the high density calculation resources of the CRIANN (www.criann.fr).

*RNA extraction and removal of genomic DNA*. Swab supernatant (150 μl) was subjected to RNA extraction using the EZ1 RNA Tissue Mini kit (QIAGEN) following the manufacturer’s instructions. Genomic DNA was then depleted from the eluate by incubation with a Turbo DNA-free kit (Thermo Fisher Scientific) according to the manufacturer’s instructions.

*Sample qualification by quantification of the SARS-CoV-2 N gene*. The N gene in the eluate was quantified by real-time reverse-transcription (RT-qPCR) using the SuperScript^TM^ III Platinum^TM^ One-Step Quantitative RT-PCR System (Invitrogen) as described previously with minor modifications [26]. Briefly, a 25 μL reaction contained 5 μL of RNA, 12.5 μL of 2 × reaction buffer, 1 μL of reverse transcriptase/Taq mixture, 0.4 μL of a 50 mM magnesium sulphate solution (Invitrogen), 1 μl of a 10 μM primer, and 0.5l μl of a 10 μM TxRd-BHQ2 probe. Thermal cycling parameters were 50°C for 15 min for reverse transcription, followed by 95°C for 2 min and then 45 cycles of 95°C for 15 s and 60°C for 30 s using a Light Cycler 480 (Roche).

*Ribosomal RNA depletion and real-time reverse-transcription PCR*. Eukaryotic rRNA was depleted using the NEBNext rRNA Depletion Kit (Human/Mouse/Rat). After rRNA depletion, cDNA was synthesized from residual total RNA by RT-VILO (Invitrogen) reaction following the manufacturer’s instructions. The material was then randomly amplified with a QuantiTect Whole Transcriptome kit (QIAGEN) according to the manufacturer’s protocol. The amplified DNA was then purified using AMPure XP beads and subjected to Qubit quantification using a dsDNA BR Assay Kit with a Qubit 3.0 fluorimeter (Invitrogen). This untargeted approach was complemented by an adapted version of the protocol published by the ARTIC Network [29] using ARTIC primer scheme version 3, which produces ~400 bp of amplicons overlapping the SARS-CoV-2 genome.

*ONT library preparation and MinION sequencing*. The libraries were not sheared so as to maximize the read length. Sequencing libraries and the sequencing reaction were carried out according to the manufacturer’s instructions with minor adaptations. Briefly, we used the NEBNext Ultra II End Repair/dATailing module (E7546S, NEB, USA) to prepare 1000 ng DNA from each sample. Native barcode adapters NBD04 were ligated in Blunt/TA Ligase Master Mix (M0367S, NEB, USA), and the resulting product was purified using AMPure XP beads before being pooled to produce a 54-μl equimass pool, itself ligated to an adapter using Native Barcoding Adapter Mix (BAM). The purified final library was loaded onto an R9.4 flowcell (FLO-MIN106, Oxford Nanopore Technologies, UK), and the run was performed on a MinION Mk1B device (ONT) for 2 hours in order to obtain more than 12 Go of raw data.

*Genome assembly*. Following the MinION sequencing run, raw data were basecalled and reads subsequently demultiplexed using the Guppy GPU basecaller / barcoder (Oxford Nanopore Technologies). Raw reads were cleaned using porechop [30] and then mapped against a custom reference for SARS-CoV-2 genome comprising four Chinese and 70 early French sequences using Bowtie2 [31] and minimap2 [32] (DOI 10.5281/zenodo.7898865). Finally, consensus genome sequences based on mapped reads (above 83% coverage with an average depth above 100 and a maximum coverage depth over 3000) were generated with bcftools consensus [33].

#### Illumina sequencing

In addition to nanopore sequencing, Illumina fastq file data were also obtained from Illumina sequencing at the French National Reference Center for respiratory viruses (Institut Pasteur Paris). The sequencer was used for basecalling and demultiplexing with the manufacturer’s software (Illumina). Reads were then trimmed and filtered using Alien trimmer [34] in order to remove adapters and bases under a quality score of q20. Mapping was performed on the same reference as mentioned above using Bowtie2 [31], and consensus (majority) genomes were extracted using bcftools [35] and compared with nanopore data. In the (rare) case of discordance, higher coverage Illumina data replaced lower coverage nanopore data. After alignment and manual verification, genome sequences were then submitted to GISAID under numbers EPI_ISL_1392906 & EPI_ISL_10036487–97.

### SARS-CoV-2 genomic datasets, genetic and phylogenetic analyses

Three main independent analyses were performed in university of Caen, one using human-derived SARS-CoV-2 genomes, another using mink-derived SARS-CoV-2 genomes and the last one combining all datasets. First, in June 2021 the raw dataset of the human SARS-CoV-2 genomes was assembled from a collection of all the genomes available in the GISAID database and filtered according to the following parameters: collection date ranging from March 1, 2020 to March 30, 2021; human genomes from France; complete genomes; high coverage and low coverage excluded (n = 42700). The resulting dataset was aligned using MAFFT [36] and optimized using in-house scripts from the virology unit’s sequencing lab at Caen University by removing redundant sequences and similar genomes without losing significant diversity (99.875% pairwise nucleotide identity cut-off, 437 genomes with a difference of at least 37 nucleotides). This diversity-optimized dataset was aligned with the twelve genomes obtained from the French mink farm and analyzed by both Maximum Likelihood (ML from PhyML, SeaView—[37]) and Bayesian phylogenetic methods (Beast—[38]). Both methods used a GTR model of evolution with gamma distribution and invariable site parameters with the coalescent constant size model as a tree prior. The likelihood ratio test and posterior probability values were used to estimate node support in ML and Bayesian methods, respectively. The Bayesian phylogenetic analysis was also enriched by using collection dates as priors and an uncorrelated relaxed clock model with lognormal distribution [39]. The Markov chain was launched for 100 million iterations on an 18- core computer using the Beast 1.10.4 suite in order to reach an effective sampling size over 200 for each statistic. The maximum credibility tree was computed from a sampling of 10,000 trees after discarding the first 1000 trees considered as a burn-in.

Second, the raw dataset of the mink SARS-CoV-2 genomes was assembled using 821 mink SARS-CoV-2 genomes collected from GISAID with the same filtering options previously used for the human SARS-CoV-2 genome collection and last updated on January 5, 2022. A diversity-optimized dataset was generated using the same method as described above for the human SARS-CoV-2 dataset. This diversity-optimized dataset (SARS-CoV-2 collected from mink—*Neovison vison*) was analyzed by the maximum likelihood phylogenetic method with the twelve genomes obtained from the French mink farm. The GTR model of evolution was used with gamma distribution and invariable site parameters. Likelihood ratio test values were calculated to estimate node support.

Thirdly, additional analyses (SNPs, amino-acid variability) used representatives (n = 9) of each main clade identified from previous analyses. Only complete genomes were conserved in this refined dataset and those with several missing data covering more than 200 contiguous nucleotides in variable loci were discarded. This last dataset was aligned with the twelve genomes sequenced in this study and subjected to ML analyses. Variable positions were extracted in order to test the existence and congruence of synapomorphic variations supporting clades and French mink SARS-CoV-2 monophyly.

#### High-throughput sequencing from fecal and swab samples

In addition to the non-specific Nanopore and Illumina sequencing of pharyngo-tracheal swabs positive for SARS-CoV-2, we undertook high-throughput sequencing (HTS) on two pharyngo-tracheal swabs (samples 61-B06-11 and 61-B12-04) and one fecal sample (61-B23-05) positive by conventional RT-PCR for the presence of a partial alphacoronavirus pol gene. The swab and fecal samples were prepared as follows for HTS.

#### Preparation of RNA samples

Viral RNA was extracted from the two swab samples subjected to HTS with a volume of 140 μL using a Qiagen Viral RNA mini kit (Qiagen, France) according to the manufacturer’s instructions. Prior to HTS, the two extracted RNA samples were checked for the presence of a partial pol gene by the conventional RT-PCR for coronaviruses using forward and reverse primers PanCoV pol 15197 (F) and PanCoV pol 15635 (R).

- 100 mg of fecal sample was placed in a Virocult tube (Sigma) containing 1 mL of stabilizing buffer, then vigorously vortexed for 30 sec at 7.0 m/s six times in a Fast Prep-24 ^TM^ 5G bead beater (MP Biomedicals Germany Gmbh, Eschwege, France) then centrifuged at 10,000 x g for 5 min. A volume of 210 μL of supernatant was transferred to three individual tubes (i.e., 70 μL/tube) for RNA extraction, each filled with 1 ml of CTAB buffer (Promega France, Charbonnières les Bains, France). The three tubes were placed in a shaking heat block at 65°C, vigorously vortexed for 1 min and mixed with 40 μL of Proteinase K Solution (Promega France, Charbonnières les Bains, France) before being incubated at 70°C for 10 min then centrifuged at 10,000g for 5 min. A volume of 300 μL of clear lysate (i.e., nine tubes) was used for RNA extraction with the Promega Maxwell RSC instrument with the Maxwell RSC PureFood GMO and Authentication kit according to the manufacturer’s instructions. RNA was pooled to form a final volume of 900 μL and stored at < -70°C. The extracted fecal RNA sample tested positive by the conventional RT-PCR for coronaviruses using forward and reverse primers PanCoV pol 15197 (F) and PanCoV pol 15635 (R).

#### Whole genome sequencing and sequence analysis

HTS was performed on the three RNA extracts after an rRNA depletion step using an rRNA depletion kit (NEB, Evry, France) according to the manufacturer’s recommendations. The RNA library was prepared for each RNA sample tested using Ion Total RNA-Seq kit v2 (Life Technologies, Carlsbad, CA, USA) and then sequenced using Ion Torrent Proton technology. The reads were cleaned with the Trimmomatic 0.36 software, followed by bioinformatics analysis as previously described [40, 41] using the GenBank Mink *Caliciviridae* reference sequence MF677852.1 for the two swab RNA samples and the Mink coronavirus 1 reference sequence MN535737.1 for the fecal RNA sample to calculate sub-sampling and final alignment.

All the sequences obtained during this study are available in GenBank: Bioproject # PRJNA881217 (sample 61-B12-04), Bioproject # PRJNA881061 (B-23-05), Biosample: SAMN30886030 (sample 61-B12-04) and Biosample (B-23-05). The consensus sequence of the full-length genome of the mink *Caliciviridae* is accessible in GenBank under accession number OP485683.

## Supporting information

S1 Fig Mink SARS-CoV-2 mutations GISAID hcov-19 nextstrain genome Nextclade analysis table. (TIF)

S2 Fig SARS-CoV-2 genomes of French mink nested within the 20A clade, together with SARS-CoV-2 from human sampled at the same period of time. (TIF)

S1 Table Mink SARS-CoV-2 Nextclade analysis. (XLSX)

S2 Table Mink SARS-CoV-2 genomic variable sites. (XLSX)

S3 Table Clade and amino acid modifications_Nextclade. (XLSX)

S4 Table GISAID_hcov-19_acknowledgement_table_2022_01_20_13. (PDF)

S5 Table GISAID_hcov-19_acknowledgement_table_2022_01_05_10. (PDF)
