# Supplementary figures and images for: Investigations into SARS-CoV-2 and other coronaviruses on mink farms in France late in the first year of the COVID-19 pandemic
Source: PLoS One. 2023 Aug 25;18(8):e0290444. doi: 10.1371/journal.pone.0290444 (PMC10456147; doi:10.1371/journal.pone.0290444)

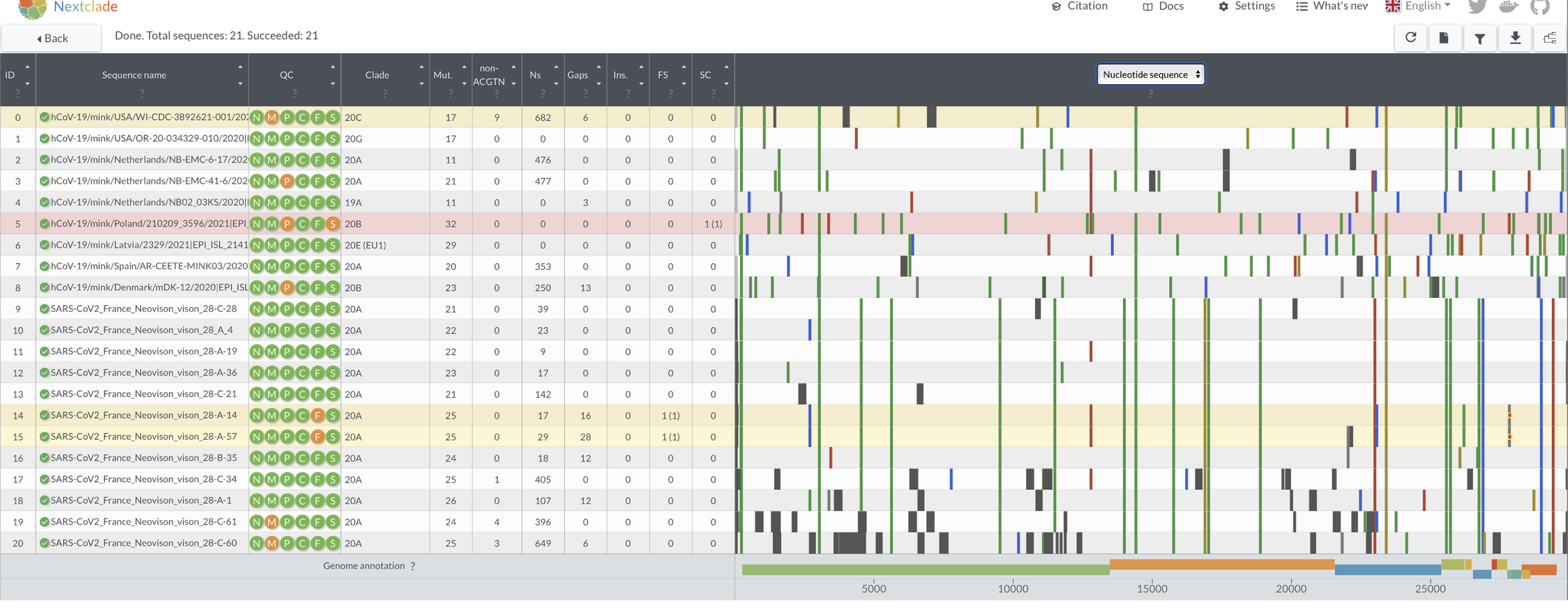

Supplement: S1 Fig — (TIF) [file pone.0290444.s001.tif]

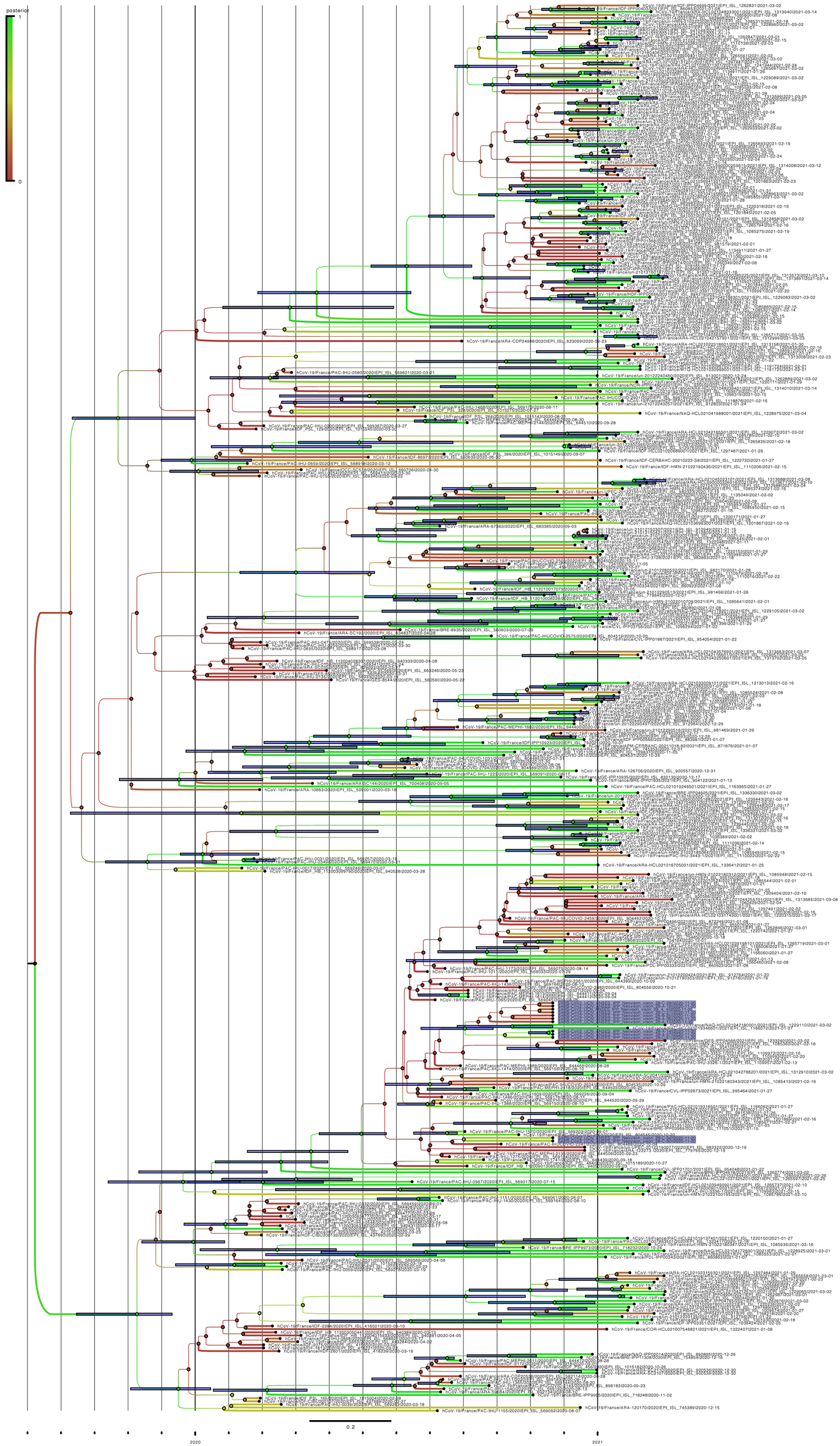

Supplement: S2 Fig — (TIF) [file pone.0290444.s002.tif]
