# Supplementary material for: Investigations into SARS-CoV-2 and other coronaviruses on mink farms in France late in the first year of the COVID-19 pandemic
Source: PLoS One. 2023 Aug 25;18(8):e0290444. doi: 10.1371/journal.pone.0290444 (PMC10456147; doi:10.1371/journal.pone.0290444)
Supplement: S4 Table — (PDF) [file pone.0290444.s006.pdf]

We gratefully acknowledge the following Authors from the Originating laboratories responsible for obtaining the specimens, as well as the Submitting laboratories where the genome data were generated and shared via GISAID, on which this research is based.

All Submitters of data may be contacted directly via www.gisaid.org

Authors are sorted alphabetically.

Accession ID	Originating Laboratory	Submitting Laboratory	Authors
EPI_ISL_717714, EPI_ISL_717715, EPI_ISL_717716, EPI_ISL_717717	Animal Health Centre, British Columbia Ministry of Agriculture	National Centre for Foreign Animal Disease, Canadian Food Inspection Agency	Asma Sultana; Brad Pickering; Oliver Lung; Peter Kruczkiewicz; Tomy Joseph
EPI_ISL_2141680, EPI_ISL_2141681, EPI_ISL_2141682, EPI_ISL_2141683, EPI_ISL_2141684, EPI_ISL_2141685, EPI_ISL_2141686, EPI_ISL_2141761, EPI_ISL_2141762, EPI_ISL_2141898, EPI_ISL_2141899, EPI_ISL_2141902, EPI_ISL_2141903, EPI_ISL_2141904, EPI_ISL_2141905, EPI_ISL_2141906, EPI_ISL_2141907, EPI_ISL_2141908, EPI_ISL_2141909, EPI_ISL_2141910, EPI_ISL_4548647, EPI_ISL_4548648, EPI_ISL_4548649, EPI_ISL_4548650, EPI_ISL_8174953, EPI_ISL_8174954, EPI_ISL_8175133, EPI_ISL_8514993, EPI_ISL_8514994, EPI_ISL_8514995, EPI_ISL_8514996, EPI_ISL_8514997, EPI_ISL_8527347, EPI_ISL_8527349, EPI_ISL_8527352			
see above	BIOR	Latvian Biomedical Research and Study Centre	Alise Jakovele; Daina Pule; Davids Fridmanis; Elina Dimina; Guntars Zarins; Irena Meistere; Ivars Silamikelis; Janis Klovins; Janis Pjalkovskis; Jurijs Perevoscikovs; Kaspars Megnis; Laila Silamikele; Lauma Freimane; Laura Ansone; Liga Birzniece; Monta Briviba; Monta Ustinova; Nikita Zrelovs; Uga Dumpis; Una Krumina; Vita Rovite
EPI_ISL_7721854	Departament of Virology National Veterinary Research Institute	Department of Omics Analysis National Veterinary Research Institute	Bomba Arkadiusz; Domanska-Blicharz Katarzyna; Iwan Ewelina; Lisowska Anna; Niemczuk Krzysztof; Opolska Justyna; Orlowska Anna; Rola Jerzy; Smreczak Marcin; Trebas Pawel
EPI_ISL_984305, EPI_ISL_984307	Department of Poultry Diseases	Department of Omics Analysis	Arkadiusz Bomba; Domanska-Blicharz Katarzyna; Ewelina Iwan; Niemczuk Krzysztof; Orlowska Anna; Smreczak Marcin
EPI_ISL_732949, EPI_ISL_732951, EPI_ISL_732952, EPI_ISL_732953, EPI_ISL_732954, EPI_ISL_732955, EPI_ISL_732956, EPI_ISL_732957, EPI_ISL_732958, EPI_ISL_732959			
see above	Department of Tropical Parasitology	Laboratory of Recombinant Vaccines	Boguslaw Szewczyk; Kirsi Aaltonen; Lukasz Rabalski; Maciej Grzybek; Maciej Kosinski; Ravi Kant; Tarja Sironen; Teemu Smura
EPI_ISL_8693816, EPI_ISL_8693906, EPI_ISL_8693911, EPI_ISL_8693912, EPI_ISL_8693913, EPI_ISL_8693914, EPI_ISL_8693915			
see above	Department of Virology, National Veterinary Research Institute	Department of Omics Analysis, National Veterinary Research Institute	Bomba Arkadiusz; Domanska-Blicharz Katarzyna; Iwan Ewelina; Lisowska Anna; Niemczuk Krzysztof; Opolska Justyna; Orlowska Anna; Rola Jerzy; Smreczak Marcin; Trebas Pawel
EPI_ISL_3218557	Department of Virology, National Veterinary Research Institute	Erasmus Medical Center	Anne van der Linden; Annemiek van der Eijk; Bas Oude Munnink; Corine GeurtsvanKessel; David Nieuwenhuijse; Domanska-Blicharz Katarzyna; Emmanuelle Munger; Irina Chestakova; Marion Koopmans; Marjan Boter; Niemczuk Krzysztof; Orlowska Anna; Reina Sikkema; Richard Molenkamp; Rola Jerzy; Smreczak Marcin; on behalf of the Dutch national COVID-19 response team.
EPI_ISL_683005, EPI_ISL_683009, EPI_ISL_683010, EPI_ISL_683014, EPI_ISL_683015, EPI_ISL_683016, EPI_ISL_683017, EPI_ISL_683018, EPI_ISL_683019, EPI_ISL_683020, EPI_ISL_683021, EPI_ISL_683022, EPI_ISL_683023, EPI_ISL_683024, EPI_ISL_683025, EPI_ISL_683026, EPI_ISL_683027, EPI_ISL_683028, EPI_ISL_683029, EPI_ISL_683030, EPI_ISL_683031, EPI_ISL_683032, EPI_ISL_683033, EPI_ISL_683034, EPI_ISL_683036, EPI_ISL_683037, EPI_ISL_683039, EPI_ISL_683041, EPI_ISL_683043, EPI_ISL_683044, EPI_ISL_683045, EPI_ISL_683047, EPI_ISL_683048, EPI_ISL_683049, EPI_ISL_683050, EPI_ISL_683051, EPI_ISL_683052, EPI_ISL_683053, EPI_ISL_683054, EPI_ISL_683055, EPI_ISL_683056, EPI_ISL_683057, EPI_ISL_683059, EPI_ISL_683061, EPI_ISL_683062, EPI_ISL_683063, EPI_ISL_683064, EPI_ISL_683065, EPI_ISL_683066, EPI_ISL_683067, EPI_ISL_683068, EPI_ISL_683069, EPI_ISL_683073, EPI_ISL_683074, EPI_ISL_683075, EPI_ISL_683076, EPI_ISL_683077, EPI_ISL_683084, EPI_ISL_683085, EPI_ISL_683086, EPI_ISL_683087, EPI_ISL_683088, EPI_ISL_683090, EPI_ISL_683091, EPI_ISL_683092, EPI_ISL_683093, EPI_ISL_683094, EPI_ISL_683095, EPI_ISL_683096, EPI_ISL_683097, EPI_ISL_683098, EPI_ISL_683099, EPI_ISL_683100, EPI_ISL_683101, EPI_ISL_683102, EPI_ISL_683103, EPI_ISL_683104, EPI_ISL_683105, EPI_ISL_683106, EPI_ISL_683108, EPI_ISL_683109, EPI_ISL_683110, EPI_ISL_683111, EPI_ISL_683112, EPI_ISL_683113, EPI_ISL_683114, EPI_ISL_683115, EPI_ISL_683116, EPI_ISL_683117, EPI_ISL_683118, EPI_ISL_683119, EPI_ISL_683120, EPI_ISL_683121, EPI_ISL_683122, EPI_ISL_683123, EPI_ISL_683124, EPI_ISL_683125, EPI_ISL_683126, EPI_ISL_683127, EPI_ISL_683128, EPI_ISL_683129, EPI_ISL_683130, EPI_ISL_683131, EPI_ISL_683132, EPI_ISL_683133, EPI_ISL_683134, EPI_ISL_683135, EPI_ISL_683136, EPI_ISL_683137, EPI_ISL_683138, EPI_ISL_683139, EPI_ISL_683140, EPI_ISL_683141, EPI_ISL_683142, EPI_ISL_683143, EPI_ISL_683144, EPI_ISL_683145, EPI_ISL_683146, EPI_ISL_683147, EPI_ISL_683148, EPI_ISL_683149, EPI_ISL_683151, EPI_ISL_683152, EPI_ISL_683153, EPI_ISL_683154, EPI_ISL_683155, EPI_ISL_683156, EPI_ISL_683157, EPI_ISL_683159, EPI_ISL_683160, EPI_ISL_683161, EPI_ISL_683162, EPI_ISL_683163, EPI_ISL_683167, EPI_ISL_683168, EPI_ISL_683169, EPI_ISL_683170, EPI_ISL_683171, EPI_ISL_683172, EPI_ISL_683173, EPI_ISL_683174, EPI_ISL_683175, EPI_ISL_683176, EPI_ISL_683177, EPI_ISL_683178, EPI_ISL_683179, EPI_ISL_683180, EPI_ISL_683181, EPI_ISL_683182, EPI_ISL_683188, EPI_ISL_683189, EPI_ISL_683192, EPI_ISL_683193, EPI_ISL_683194, EPI_ISL_683195, EPI_ISL_683196, EPI_ISL_683200, EPI_ISL_683201, EPI_ISL_683207, EPI_ISL_683208, EPI_ISL_683210, EPI_ISL_683211, EPI_ISL_683215, EPI_ISL_683216, EPI_ISL_683217, EPI_ISL_683218, EPI_ISL_683221, EPI_ISL_683222, EPI_ISL_683225, EPI_ISL_683226, EPI_ISL_683227, EPI_ISL_683229, EPI_ISL_683230, EPI_ISL_683231, EPI_ISL_683237, EPI_ISL_683239, EPI_ISL_683241, EPI_ISL_683243, EPI_ISL_683246, EPI_ISL_683247, EPI_ISL_683248, EPI_ISL_683249, EPI_ISL_683250, EPI_ISL_683251, EPI_ISL_683268, EPI_ISL_683270, EPI_ISL_683271, EPI_ISL_683272, EPI_ISL_683273, EPI_ISL_683275, EPI_ISL_683276, EPI_ISL_683279, EPI_ISL_683280, EPI_ISL_683281, EPI_ISL_683282, EPI_ISL_683283, EPI_ISL_683288, EPI_ISL_683289, EPI_ISL_683297, EPI_ISL_683298, EPI_ISL_683299, EPI_ISL_683300, EPI_ISL_683301, EPI_ISL_683302, EPI_ISL_683303, EPI_ISL_683306, EPI_ISL_683307, EPI_ISL_683308, EPI_ISL_683309, EPI_ISL_683310, EPI_ISL_683311, EPI_ISL_683312, EPI_ISL_683313, EPI_ISL_683316, EPI_ISL_683317, EPI_ISL_683318, EPI_ISL_683319, EPI_ISL_683320, EPI_ISL_683321, EPI_ISL_683322, EPI_ISL_683323, EPI_ISL_683324, EPI_ISL_683325, EPI_ISL_683326			
see above	Department of Virus and Microbiological Special Diagnostics, Statens Serum Institut, Copenhagen, Denmark	Albertsen Lab, Department of Chemistry and Bioscience, Aalborg University, Denmark	Danish Covid-19 Genome Consortium
EPI_ISL_641396, EPI_ISL_641397, EPI_ISL_641398, EPI_ISL_641399, EPI_ISL_641400, EPI_ISL_641401, EPI_ISL_641402, EPI_ISL_641403, EPI_ISL_641404, EPI_ISL_641405, EPI_ISL_641406, EPI_ISL_641407, EPI_ISL_641408, EPI_ISL_641409, EPI_ISL_641410, EPI_ISL_641411, EPI_ISL_641412, EPI_ISL_641413, EPI_ISL_641414, EPI_ISL_641415, EPI_ISL_641416, EPI_ISL_641419, EPI_ISL_641420, EPI_ISL_641421, EPI_ISL_641422, EPI_ISL_641423, EPI_ISL_641428, EPI_ISL_641433, EPI_ISL_641435, EPI_ISL_641438, EPI_ISL_641439, EPI_ISL_641440, EPI_ISL_641441, EPI_ISL_641442, EPI_ISL_641444, EPI_ISL_641445, EPI_ISL_641446, EPI_ISL_641447, EPI_ISL_641449, EPI_ISL_641450, EPI_ISL_641451, EPI_ISL_641452, EPI_ISL_641454, EPI_ISL_641455, EPI_ISL_641457, EPI_ISL_641458, EPI_ISL_641459, EPI_ISL_641460, EPI_ISL_641462, EPI_ISL_641463, EPI_ISL_641464, EPI_ISL_641465, EPI_ISL_641466, EPI_ISL_641467, EPI_ISL_641468, EPI_ISL_641470, EPI_ISL_641471, EPI_ISL_641472, EPI_ISL_641473, EPI_ISL_641474, EPI_ISL_641475, EPI_ISL_641476, EPI_ISL_641477, EPI_ISL_641478, EPI_ISL_641479, EPI_ISL_641480, EPI_ISL_641481, EPI_ISL_641482, EPI_ISL_641483, EPI_ISL_641484, EPI_ISL_641485, EPI_ISL_641486, EPI_ISL_641487, EPI_ISL_641488, EPI_ISL_641489, EPI_ISL_641490, EPI_ISL_641491, EPI_ISL_641492, EPI_ISL_641493, EPI_ISL_641494, EPI_ISL_641495, EPI_ISL_641496, EPI_ISL_641497, EPI_ISL_641498, EPI_ISL_641500, EPI_ISL_641501, EPI_ISL_641502, EPI_ISL_641503, EPI_ISL_641504, EPI_ISL_641505, EPI_ISL_641506, EPI_ISL_641507, EPI_ISL_641508, EPI_ISL_641509, EPI_ISL_641510, EPI_ISL_641511, EPI_ISL_641512, EPI_ISL_641513, EPI_ISL_641516, EPI_ISL_3050753, EPI_ISL_3050754, EPI_ISL_3050755, EPI_ISL_3050756, EPI_ISL_3050757, EPI_ISL_3050760, EPI_ISL_3050761, EPI_ISL_3050762, EPI_ISL_3050763, EPI_ISL_3050764, EPI_ISL_3050765			
see above	Department of Virus and Microbiological Special Diagnostics, Statens Serum Institut, Copenhagen, Denmark	Albertsen lab, Department of Chemistry and Bioscience, Aalborg University, Denmark	Danish Covid-19 Genome Consortia; Jannik Fonager; Morten Rasmussen; Thomas Bruun Rasmussen
EPI_ISL_522987, EPI_ISL_522988, EPI_ISL_522989, EPI_ISL_522990, EPI_ISL_522992, EPI_ISL_522994, EPI_ISL_522995, EPI_ISL_522996, EPI_ISL_522997, EPI_ISL_522998, EPI_ISL_522999, EPI_ISL_523000, EPI_ISL_523001, EPI_ISL_523002, EPI_ISL_523003, EPI_ISL_523004, EPI_ISL_523005, EPI_ISL_523006, EPI_ISL_523007, EPI_ISL_523008, EPI_ISL_523009, EPI_ISL_523010, EPI_ISL_523012, EPI_ISL_523014, EPI_ISL_523015, EPI_ISL_523016, EPI_ISL_523017, EPI_ISL_523018, EPI_ISL_523019, EPI_ISL_523020, EPI_ISL_523022, EPI_ISL_523023, EPI_ISL_523024, EPI_ISL_523025, EPI_ISL_523026, EPI_ISL_523027, EPI_ISL_523028, EPI_ISL_523029, EPI_ISL_523030, EPI_ISL_523031, EPI_ISL_523032, EPI_ISL_523033, EPI_ISL_523034, EPI_ISL_523035, EPI_ISL_523036, EPI_ISL_523037, EPI_ISL_523038, EPI_ISL_523039, EPI_ISL_523041, EPI_ISL_523042, EPI_ISL_523043, EPI_ISL_523044, EPI_ISL_523045, EPI_ISL_523046, EPI_ISL_523048, EPI_ISL_523049, EPI_ISL_523050, EPI_ISL_523051, EPI_ISL_523052, EPI_ISL_523053, EPI_ISL_523054, EPI_ISL_523055, EPI_ISL_523056, EPI_ISL_523057, EPI_ISL_523058, EPI_ISL_523059, EPI_ISL_523060, EPI_ISL_523061, EPI_ISL_523062, EPI_ISL_523063, EPI_ISL_523064, EPI_ISL_523065, EPI_ISL_523066, EPI_ISL_523067, EPI_ISL_523069, EPI_ISL_523070, EPI_ISL_523071, EPI_ISL_523074, EPI_ISL_523075, EPI_ISL_523076, EPI_ISL_523077, EPI_ISL_523079, EPI_ISL_523080, EPI_ISL_523081, EPI_ISL_523082, EPI_ISL_523083, EPI_ISL_523084, EPI_ISL_523085, EPI_ISL_523086, EPI_ISL_523087, EPI_ISL_523091, EPI_ISL_523092, EPI_ISL_523093, EPI_ISL_523094, EPI_ISL_523095, EPI_ISL_523096, EPI_ISL_523097, EPI_ISL_523098, EPI_ISL_523099, EPI_ISL_523100, EPI_ISL_523101, EPI_ISL_523103, EPI_ISL_523104, EPI_ISL_523105, EPI_ISL_523106, EPI_ISL_523108, EPI_ISL_523109, EPI_ISL_523110, EPI_ISL_523111, EPI_ISL_523112, EPI_ISL_523113, EPI_ISL_523114, EPI_ISL_523115, EPI_ISL_523116, EPI_ISL_523117, EPI_ISL_523118, EPI_ISL_523119, EPI_ISL_577749, EPI_ISL_577750, EPI_ISL_577752, EPI_ISL_577753, EPI_ISL_577754, EPI_ISL_577755, EPI_ISL_577756, EPI_ISL_577758, EPI_ISL_577764, EPI_ISL_577767, EPI_ISL_577768, EPI_ISL_577770, EPI_ISL_577771, EPI_ISL_577772, EPI_ISL_577773, EPI_ISL_577774, EPI_ISL_577775, EPI_ISL_577776, EPI_ISL_577781, EPI_ISL_577783, EPI_ISL_577784, EPI_ISL_577785, EPI_ISL_577786, EPI_ISL_577787, EPI_ISL_577791, EPI_ISL_577797, EPI_ISL_577798, EPI_ISL_577799, EPI_ISL_577800, EPI_ISL_577801, EPI_ISL_577802, EPI_ISL_577803, EPI_ISL_577805, EPI_ISL_577808, EPI_ISL_577809, EPI_ISL_577810, EPI_ISL_577813, EPI_ISL_577825, EPI_ISL_632428, EPI_ISL_632429, EPI_ISL_632430, EPI_ISL_632434, EPI_ISL_632435, EPI_ISL_632436, EPI_ISL_632437, EPI_ISL_632438, EPI_ISL_632439, EPI_ISL_632450, EPI_ISL_632451, EPI_ISL_632452, EPI_ISL_632453, EPI_ISL_632454, EPI_ISL_632455, EPI_ISL_632456, EPI_ISL_632457, EPI_ISL_632458, EPI_ISL_632459, EPI_ISL_632462, EPI_ISL_632463, EPI_ISL_632464, EPI_ISL_632465, EPI_ISL_632466, EPI_ISL_632467, EPI_ISL_632468, EPI_ISL_632469, EPI_ISL_632470, EPI_ISL_632471, EPI_ISL_632472, EPI_ISL_632473, EPI_ISL_632474, EPI_ISL_632475, EPI_ISL_632476, EPI_ISL_632477, EPI_ISL_632478, EPI_ISL_632483, EPI_ISL_632484, EPI_ISL_632485, EPI_ISL_632486, EPI_ISL_632487, EPI_ISL_632488, EPI_ISL_632489, EPI_ISL_632490, EPI_ISL_632491, EPI_ISL_632492, EPI_ISL_722414, EPI_ISL_722415, EPI_ISL_722416, EPI_ISL_722417, EPI_ISL_722418, EPI_ISL_722448, EPI_ISL_722449, EPI_ISL_722450, EPI_ISL_722451, EPI_ISL_904320, EPI_ISL_904321, EPI_ISL_904322, EPI_ISL_904323, EPI_ISL_904324, EPI_ISL_904325, EPI_ISL_904326, EPI_ISL_904327, EPI_ISL_904328, EPI_ISL_904329, EPI_ISL_904330, EPI_ISL_904331, EPI_ISL_904332, EPI_ISL_904333, EPI_ISL_904334, EPI_ISL_904335, EPI_ISL_904336, EPI_ISL_904337, EPI_ISL_904338, EPI_ISL_904339, EPI_ISL_904340, EPI_ISL_904341, EPI_ISL_904342, EPI_ISL_904343, EPI_ISL_904344, EPI_ISL_904345, EPI_ISL_904346, EPI_ISL_904347, EPI_ISL_904348, EPI_ISL_904349, EPI_ISL_904350, EPI_ISL_904351, EPI_ISL_904352, EPI_ISL_904353, EPI_ISL_904354, EPI_ISL_904355, EPI_ISL_904356, EPI_ISL_904357, EPI_ISL_904358, EPI_ISL_904359, EPI_ISL_904360, EPI_ISL_904625			
see above	Dutch COVID-19 response team	Erasmus Medical Center	Anne van der Linden; Annemiek van der Eijk; Bas Oude Munnink; Corine GeurtsvanKessel; David Nieuwenhuijse; Emmanuelle Munger; Irina Chestakova; Marion Koopmans; Marjan Boter; OH consortium; Reina Sikkema; Richard Molenkamp; on behalf of the OH consortium.
EPI_ISL_1265354, EPI_ISL_1265355, EPI_ISL_1265356, EPI_ISL_1265357, EPI_ISL_1265358, EPI_ISL_1265359, EPI_ISL_1265360, EPI_ISL_1265361, EPI_ISL_1265362, EPI_ISL_1265363, EPI_ISL_1265364, EPI_ISL_1265365, EPI_ISL_1265366, EPI_ISL_1265367			
see above	Dutch COVID-19 response team	Wageningen Bioveterinary Research	OH consortium
EPI_ISL_8144460, EPI_ISL_8144462	E. Gulbja Laboratorija	Latvian Biomedical Research and Study Centre	Alise Jakovele; Davids Fridmanis; Dmitrijs Perminovs; Elina Dimina; Guntars Zarins; Ivars Silamikelis; Janis Klovins; Janis Pjalkovskis; Jurijs Perevoscikovs; Kaspars Megnis; Laila Silamikele; Lauma Freimane; Laura Ansone; Liga Birzniece; Mikus Gavars; Monta Briviba; Nikita Zrelovs; Uga Dumpis; Una Krumina; Vita Rovite
EPI_ISL_1392906	Laboratoire de la rage et de la faune sauvage, ANSES, Malzéville.	GRAM2.0, Université de Caen Normandie / Laboratoire de Virologie, CHU de Caen / National Reference Center for Respiratory Viruses France Nord, Institut Pasteur, Paris. / Laboratoire de la rage et de la faune sauvage ANSES, Malzéville.	Angela BRISEBARRE; Astrid VABRET; Camille Capel; Elodie MONCHATRE-LEROY.; Estelle LEPERCHOIS; Etienne SIMON-LORIERE; Evelyne PICARD-MEYER; Fabiana GAMBARO; Flora DONATI; Franck BOUE; Gilles SALVAT; Januel VOUTEAU; Justine HAMEL; Marine WASNIEWSKY; Maud Vanpeene; Meriadeg AR GOUILH; Mélanie ALBERT; Méline Bizard; Nicolas NESI; Sandrine CORBET; Sophie LEVALTIER; Sylvie BEHILLIL; Sylvie VAN DER WERF; Vincent ENOUF
EPI_ISL_925309, EPI_ISL_925310, EPI_ISL_925311, EPI_ISL_925312, EPI_ISL_925313, EPI_ISL_925314, EPI_ISL_925315, EPI_ISL_925316, EPI_ISL_925317, EPI_ISL_925318, EPI_ISL_925319, EPI_ISL_925320, EPI_ISL_925321, EPI_ISL_925322, EPI_ISL_925323, EPI_ISL_925324, EPI_ISL_925325, EPI_ISL_925326, EPI_ISL_925327, EPI_ISL_925328, EPI_ISL_925329, EPI_ISL_925330, EPI_ISL_925331, EPI_ISL_925332, EPI_ISL_925333, EPI_ISL_925334, EPI_ISL_925335, EPI_ISL_925336, EPI_ISL_925337, EPI_ISL_925338, EPI_ISL_925339, EPI_ISL_925340, EPI_ISL_925341, EPI_ISL_925342, EPI_ISL_925343, EPI_ISL_925344, EPI_ISL_925345, EPI_ISL_925346, EPI_ISL_925347, EPI_ISL_925348, EPI_ISL_925349, EPI_ISL_925350, EPI_ISL_925351, EPI_ISL_925352, EPI_ISL_925353, EPI_ISL_925354, EPI_ISL_925355, EPI_ISL_925356, EPI_ISL_925357, EPI_ISL_925358, EPI_ISL_925359, EPI_ISL_925360			
see above	Michigan Department of Agriculture and Rural Development	Pathogen Discovery, Respiratory Viruses Branch, Division of Viral Diseases, Centers for Disease Control and Prevention	Anna Uehara; Anna montmayeur; Brian Lynch; Clinton R. Paden; Haibin Wang; Jing Zhang; Krista Queen; Peter Cook; Rachel Marine; Suxiang Tong; Yan Li; Ying Tao
EPI_ISL_2834696, EPI_ISL_2834697	Michigan State University Veterinary Diagnostic Laboratory	Diagnostic Virology Laboratory, National Veterinary Services Laboratories, USDA1920 Dayton Avenue, Ames, IA 50010, USA	Emily R. Love; Kerrie M. Franzen; Mary L. Killian; Mia Torchetti; Suelee Robbe-Austerman; Tod P. Stuber
EPI_ISL_851055, EPI_ISL_851056, EPI_ISL_851057, EPI_ISL_851058, EPI_ISL_851059, EPI_ISL_851060, EPI_ISL_851061			
see above	National Food and Veterinary Risk Assessment Institute	Lithuanian University of Health Sciences, Molecular cardiology lab.	Algirdas Malakauskas; Arnoldas Pautienius; Arunas Stankevicius; Astra Vitkauskiene; Dovydas Gecys; Ingrida Olendraitė; Kamile Tamusauskaite; Laura Pareckaite; Lukas Zemaitis; Vaiva Lesauskaite
EPI_ISL_1527630, EPI_ISL_1527631, EPI_ISL_1527632, EPI_ISL_1527633	Oregon Department of Agriculture	Diagnostic Virology Laboratory, National Veterinary Services Laboratories, USDA1920 Dayton Avenue, Ames, IA 50010, USA	Emily R. Love; Kerrie M. Franzen; Mary L. Killian; Mia Torchetti; Suelee Robbe-Austerman; Tod P. Stuber
EPI_ISL_626340, EPI_ISL_626341, EPI_ISL_626342, EPI_ISL_626343, EPI_ISL_626344, EPI_ISL_626345, EPI_ISL_626346, EPI_ISL_626347, EPI_ISL_626348, EPI_ISL_626349, EPI_ISL_626350, EPI_ISL_626351			
see above	Statens Serum Institute	Statens Serum Institute	A.S.; Afaro-Nunez, A.; Belsham; C.S.; Fonager, J.; Foomsgaard, A.; G.J. and Botner, A.; Halasa, T.; Hammer; Jorgensen; Lohse, L.; M.L.; M.W.; Mundbjerg, K.; Quaade; Rasmussen; Rasmussen, M.; Rosenstierne; Strandbygaard, B.; T.B.
EPI_ISL_2467943	The Laboratory of Biosafety with Pathogens Collection The Republican Research and Practical Center for Epidemiology and Microbiology (RRPCEM)	Laboratory for HIV and opportunistic infections diagnosis The Republican Research and Practical Center for Epidemiology and Microbiology (RRPCEM)	Anatoli Krasko; Artur Akhremchuk; Elena Gasich; Ilarion Kupryianav; Iryna Subotsina; Kirill Bulda; Larysa Radzienava; Leonid Valentovich; Olga Klimovich; Olga Saleuskaya; Sergei Semenov
EPI_ISL_2834689, EPI_ISL_2834690, EPI_ISL_2834691, EPI_ISL_2834692, EPI_ISL_2834693, EPI_ISL_2834694, EPI_ISL_2834695, EPI_ISL_2834937, EPI_ISL_2834938, EPI_ISL_2834939, EPI_ISL_2834940, EPI_ISL_2834941, EPI_ISL_2834942, EPI_ISL_2834943, EPI_ISL_2834944, EPI_ISL_2834945, EPI_ISL_2896207, EPI_ISL_2896208, EPI_ISL_2896209, EPI_ISL_2896210, EPI_ISL_2896211, EPI_ISL_2896212, EPI_ISL_2896213			
see above	Utah Veterinary Diagnostic Laboratory	Diagnostic Virology Laboratory, National Veterinary Services Laboratories, USDA1920 Dayton Avenue, Ames, IA 50010, USA	Emily R. Love; Kerrie M. Franzen; Mary L. Killian; Mia Torchetti; Suelee Robbe-Austerman; Tod P. Stuber
EPI_ISL_431778, EPI_ISL_447626, EPI_ISL_447627, EPI_ISL_447628, EPI_ISL_447629, EPI_ISL_447630, EPI_ISL_447631, EPI_ISL_447632, EPI_ISL_447634			
see above	Virology, Wageningen Bioveterinary Research	Virology, Wageningen Bioveterinary Research	A. and van der Poel; Bouwstra, R.; Engelsma; Gerhards, N.; Hakze van der Honing; Harders, F.; Hissink, H.; M.Y.; Molenaar; Oreshkova, N.; R.J.; R.W.; Smit, L.; Stegeman; Stegeman, A.; Tacken, M.; Vreman, S.; W.H.M.; Weesendorp, E.; van der Poel; van der Poel, W.
EPI_ISL_2896203, EPI_ISL_2896204, EPI_ISL_2896205, EPI_ISL_2896206	Washington Animal Disease Diagnostic Laboratory	Diagnostic Virology Laboratory, National Veterinary Services Laboratories, USDA1920 Dayton Avenue, Ames, IA 50010, USA	Emily R. Love; Kerrie M. Franzen; Mary L. Killian; Mia Torchetti; Suelee Robbe-Austerman; Tod P. Stuber
EPI_ISL_1014949, EPI_ISL_1014950, EPI_ISL_1014951, EPI_ISL_1014952, EPI_ISL_1014953, EPI_ISL_1014954, EPI_ISL_1014955, EPI_ISL_1014956, EPI_ISL_1014957, EPI_ISL_1014958, EPI_ISL_1014959, EPI_ISL_1014960, EPI_ISL_1014961, EPI_ISL_1014962, EPI_ISL_1014963, EPI_ISL_1014964, EPI_ISL_1014965, EPI_ISL_1014966, EPI_ISL_1014967, EPI_ISL_1014968, EPI_ISL_1014969, EPI_ISL_1014970, EPI_ISL_1014971, EPI_ISL_1014972, EPI_ISL_1014973, EPI_ISL_1014974, EPI_ISL_1014975, EPI_ISL_1014976, EPI_ISL_1014977, EPI_ISL_1014978, EPI_ISL_1014979, EPI_ISL_1014980, EPI_ISL_1014981, EPI_ISL_1014982, EPI_ISL_1014983, EPI_ISL_1014984, EPI_ISL_1014985, EPI_ISL_1014986, EPI_ISL_1014987			
see above	Wisconsin Department of Agriculture, Trade, and Consumer	Pathogen Discovery, Respiratory Viruses Branch, Division of Viral Diseases, Centers for Disease	Anna Montmayeur; Anna Uehara; Brian Lynch; Clinton R. Paden; Haibin Wang; Han Jia Ng; Jing Zhang; Krista Queen; Peter Cook; Rachel Marine; Suxiang Tong; Yan Li; Ying Tao

	Protection	Control and Prevention	
EPI_ISL_2834698, EPI_ISL_2834699, EPI_ISL_2834700, EPI_ISL_2834701, EPI_ISL_2834702	Wisconsin Veterinary Diagnostic Laboratory	Diagnostic Virology Laboratory, National Veterinary Services Laboratories, USDA1920 Dayton Avenue, Ames, IA 50010, USA	Emily R. Love; Kerrie M. Franzen; Mary L. Killian; Mia Torchetti; Suelee Robbe-Austerman; Tod P. Stuber
