# Supplementary material for: Investigations into SARS-CoV-2 and other coronaviruses on mink farms in France late in the first year of the COVID-19 pandemic
Source: PLoS One. 2023 Aug 25;18(8):e0290444. doi: 10.1371/journal.pone.0290444 (PMC10456147; doi:10.1371/journal.pone.0290444)
Supplement: S5 Table — (PDF) [file pone.0290444.s007.pdf]

We gratefully acknowledge the following Authors from the Originating laboratories responsible for obtaining the specimens, as well as the Submitting laboratories where the genome data were generated and shared via GISAID, on which this research is based.

All Submitters of data may be contacted directly via www.gisaid.org

Authors are sorted alphabetically.

Accession ID	Originating Laboratory	Submitting Laboratory	Authors
EPI_ISL_5781754	Bronson Animal Disease Diagnostic Laboratory	Diagnostic Virology Laboratory, National Veterinary Services Laboratories, USDA1920 Dayton Avenue, Ames, IA 50010, USA	Emily R. Love; Kerrie M. Franzen; Mary L. Killian; Mia Torchetti; Suelee Robbe-Austerman; Tod P. Stuber
EPI_ISL_2521999	The Laboratory of Biosafety with Pathogens Collection The Republican Research and Practical Center for Epidemiology and Microbiology (RRPCEM)	Laboratory for HIV and opportunistic infections diagnosis The Republican Research and Practical Center for Epidemiology and Microbiology (RRPCEM)	Anatoli Krasko; Artur Akhremchuk; Elena Gasich; Ilarion Kupryianav; Iryna Subotsina; Kirill Bulda; Larysa Radzienava; Leonid Valentovich; Olga Klimovich; Olga Saleuskaya; Sergei Semenov
EPI_ISL_1490186	University of Ljubljana, Faculty of Veterinary Medicine	Institute of Microbiology and Immunology, Faculty of Medicine, University of Ljubljana	Ana Kočevar; Brigita Slavec; Jožko Račnik; Mario Poljak; Matic Brvar; Milan Matko; Miša Korva; Olga Zorman Rojs; Samo Zakotnik; Tatjana Avšič – Županc; Tomaž Mark Zorec
